# How effective is virtual reality technology in palliative care? A systematic review and meta-analysis

**DOI:** 10.1177/02692163221099584

**Published:** 2022-05-30

**Authors:** Jiping Mo, Victoria Vickerstaff, Ollie Minton, Simon Tavabie, Mark Taubert, Patrick Stone, Nicola White

**Affiliations:** 1 UCL Division of Psychiatry, London, UK; 2 Marie Curie Palliative Care Research Department, UCL Division of Psychiatry, London, UK; 3 Priment Clinical Trials Unit, Research Department of Primary Care and Population Health, University College London (UCL), London, UK; 4 Sussex Cancer Centre University Hospitals, Sussex, UK; 5 St Joseph’s Hospice, Hackney, UK; 6 Palliative Medicine, Velindre Cancer Centre, Cardiff, UK; 7 Palliative Care, Cardiff University School of Medicine, Cardiff, UK

**Keywords:** Palliative care, virtual reality, technology, electronics, medical

## Abstract

**Background:**

The efficacy of virtual reality for people living with a terminal illness is unclear.

**Aim:**

To determine the feasibility and effectiveness of virtual reality use within a palliative care setting.

**Design:**

Systematic review and meta-analysis. PROSPERO (CRD42021240395).

**Data sources:**

Medline, Embase, AMED, PsycINFO, CINAHL, Cochrane Central Register of Controlled Trials and Web of Science were searched from inception to March 2021. Search terms included ‘virtual reality’ and ‘palliative care’. Eligibility: (1) adult (>18 years old) with a terminal illness (2) at least one virtual reality session and (3) feasibility data and/or at least one patient outcome reported. The ROB-2 and ROBINS tools assessed risk of bias. The Grading of Recommendations, Assessment, Development and Evaluations (GRADE) tool assessed the quality of the evidence. Standardised mean differences (Hedges’s *g*) were calculated from the pre- and post-data. A DerSimonian-Laird random effects model meta-analysis was conducted.

**Results:**

Eight studies were included, of which five were in the meta-analysis. All studies had at least some concern for risk of bias. Virtual reality statistically significantly improved pain (*p* = 0.0363), tiredness (*p* = 0.0030), drowsiness (*p* = 0.0051), shortness of breath (*p* = 0.0284), depression (*p* = 0.0091) and psychological well-being (*p* = 0.0201). The quality of the evidence was graded as very low due to small sample sizes, non-randomisation methods and a lack of a comparator arm.

**Conclusions:**

Virtual reality in palliative care is feasible and acceptable. However, limited sample sizes and very low-quality studies mean that the efficacy of virtual reality needs further research.

**What is already known on this topic?**

- Virtual reality is available as a technology in clinical practice without specific indications or measurement of clinical benefit.
- There is limited evidence as to the efficacy of its use within a palliative population.

**What this paper adds?**

- This review highlights the limited and often very low-quality evidence about efficacy of virtual reality in palliative care.
- The data from this review suggests that the technology is generally well tolerated with some possible therapeutic potential.

**Implications for practice, theory or policy**

- This review highlights the methodological and clinical challenges that need to be addressed in order to fully understand the efficacy of virtual reality in a palliative care setting. Higher quality and larger studies, with a comparator arm, exploring the use of virtual reality in palliative care settings is critical.

## Introduction

Hand-held technology has rapidly improved to become one of the main methods of communication and accessing information in daily life. Prior to COVID-19, healthcare services were already being digitalised.^1^ The public were becoming more familiar with using different technology as part of their routine healthcare – be that the management of an illness (e.g. diabetes) or by video calling a primary care provider. Since the COVID-19 pandemic, digitalisation of healthcare will continue and it is important to understand the future applications as well as benefits and possible harms.

The specific technology focus of this review is virtual reality. Over the last two decades, it has become more portable and accessible (in terms of cost and availability). Large technology companies such as Google and Facebook have invested in virtual reality and are currently developing better virtual reality equipment and platforms. More pertinently to healthcare, virtual reality has been used to help train surgeons to operate by visualising the complex vascular supplies around tumours,^2^ and in simulations around end-of-life care.^3^ Virtual reality immerses the individual in a three-dimensional world (with experiences such as underwater diving, rollercoasters) often by using a headset, sometimes with handheld remotes. This immersion experience can trigger similar physical and emotional responses akin to being physically in the location being viewed^4^; for this reason, the therapeutic benefit of virtual reality has been researched.

There have been four Cochrane reviews investigating the potential therapeutic benefits of virtual reality: in paediatric pain,^5^ rehabilitation following a stroke^6^ and for people with Parkinson Disease,^7^ also in treatment compliance for serious mental illness.^8^ All reported that it was difficult to make recommendations for clinical practice due to low quality studies and low strength of evidence; all advocated for larger trials. Multiple systematic reviews have also been conducted exploring the efficacy of virtual reality in different settings. Pain is a common symptom that has been addressed in virtual reality research; in paediatric populations,^9^ adult populations^10,11^ and during specific interventions or procedures.^12–14^ Two systematic reviews looked more globally at the effect of virtual reality on common physical and mental health issues in any setting (e.g. anxiety, pain, depression).^15,16^

There has been a mini-review looking at the evidence of virtual reality for people living with dementia^17^ and for people undergoing cancer treatment^18^ however, to date, there has been no review to date that has evaluated the effectiveness of virtual reality specifically in a palliative care population. People living with a terminal illness often have multiple and complex physical and mental health needs^19,20^ and virtual reality may have a role as an adjuvant non-pharmacological contribution to the management of complex symptoms. This review aims to determine the extent of the evidence regarding the efficacy of virtual reality within palliative care. Due to the novel nature of the technology, the review focuses on the feasibility and acceptability of the technology, as well as identifying reported physical and psychological effects.

## Methods

The protocol for this review was registered prospectively with PROSPERO (CRD42021240395, 3rd March 2021). This review was conducted using the Cochrane handbook for conducting the systematic reviews^21^ and reported following the Preferred Reporting Items for Systematic Reviews and Meta-Analyses (PRISMA) guideline.^22^ Ethical approval was not required for this review.

### Aim

The overall aim of the review was to determine the feasibility and effectiveness of virtual reality use within a palliative care setting.

The objectives were:

1. To describe the virtual reality technology that has been used in a palliative care setting.
2. To describe the feasibility and acceptability of the technology.
3. To explore the efficacy of virtual reality in a palliative care setting.

### Eligibility criteria

Studies were included if they reported on the use of virtual reality in a palliative population. To define this population, we included any study that described the participant group as having an illness that was no longer curative or not receiving curative treatment; synonyms of this included ‘end of life’, ‘palliative’ and ‘terminal’.

#### Inclusion criteria

1. Human adults (over 18 years of age).
2. Palliative participant group (or a synonym of palliative, i.e. ‘not curable’, ‘terminal’, ‘stage 4’).
3. Participants completed at least one virtual reality session.
4. Outcome measures reported included at least one of the following: feasibility, acceptability efficacy (through a validated measure) on physical and/or psychological symptoms.
5. Randomised Control Trial (RCT), a non-RCT or a pre-post design.
6. English language.

Studies that were solely qualitative were excluded from this review. Mixed method studies were included as long as they met the criteria above. Studies were excluded if they did not meet the inclusion criteria.

### Data sources

The following electronic databases were searched from inception up until 26th March 2021: Medline (OVID), Embase (OVID), AMED (OVID), PsycINFO (OVID), CINAHL (EBSCOhost), Cochrane Central Register of Controlled Trials (CENTRAL) and Web of Science.

### Search strategy

The search strategy was developed in consultation with a specialist librarian at the University College London. Search terms combined two concepts: (1) ‘Palliative care’ and (2) ‘Virtual reality’. Relevant key concepts were identified from a previous review in palliative care,^23^ a recent Cochrane review^5^ and searched using Mesh terms in PubMed and equivalent terms in other databases, with tailored searches being developed for each database (see e.g. search strategy in Supplemental Material 1). The search strategy was piloted and refined, particularly the search terms for ‘virtual reality’, initially to balance sensitivity (retrieving a high number of relevant articles) and specificity (retrieving a low number of irrelevant articles) of searches. In addition to searching the databases, the lead author (JM) also screened the reference lists of included papers for relevant articles. JM also contacted the authors of the included studies for any unreported data, unpublished or ongoing work.

### Selection process

At the first stage of screening, two reviewers (NW and JM) independently reviewed the titles and abstracts of studies identified from the database searches. Reviewers screened against the following criteria: (1) the study reported using virtual reality technology and (2) participants were described as receiving palliative care. For the second stage, the same two reviewers conducted full-text screening. Reviewers screened against the full inclusion criteria (see Inclusion criteria). At both stages of screening, any disagreement on included studies was resolved by liaising a third reviewer (PS).

### Data collection process

The research team developed a data extraction form to code the demographic, methodological and outcome variables extracted from each study, with data extraction performed by NW and JM independently.

### Data extraction

Final data to be included in the analysis were confirmed by both NW and JM. Information on study characteristics were extracted, including authors, country, year, sample size, design, setting, recruitment. Participant characteristics were extracted including age, gender, diagnosis. virtual reality characteristics were also extracted, including virtual reality type, dosing, comparison group, virtual reality results (feasibility, acceptability, efficacy). Feasibility and acceptability data were extracted for all included studies, while efficacy data were extracted for studies that reported quantitative data using validated measures.

### Risk of bias assessment

Two reviewers (NW and JM) independently assessed the quality of the included studies, and any disagreement was resolved through revision and discussion. The Cochrane risk-of-bias tool for randomised trials version 2 (RoB 2)^24^ was used to assess the quality of RCTs. RoB 2 contains five domains of bias: randomisation, deviations from the intended interventions, missing outcome data, measurement of the outcome and reporting results. Judgement about the risk of bias for each domain was either ‘Low’, ‘Some concerns’, ‘High’ risk of bias. Non-randomised control trials were assessed using the Cochrane Risk of Bias in non-randomised studies – of Interventions (ROBIN-I).^25^ The tool contains seven domains: confounding, participant selection, classification of intervention, deviations from the intended interventions, missing data, measurement of outcomes and reporting results. Judgement for each domain was rated as either ‘Low’, ‘Moderate’, ‘Serious’ and ‘Critical’. An overall risk of bias judgement was made based on judgement for the seven individual domains.

No study was excluded based on their quality score, but they are reported for transparency.

### Data synthesis and analysis

A summary of the study characteristics (e.g. study design, setting), demographics of the patient population (e.g. age, gender, diagnosis) and details about the delivery of virtual reality (e.g. frequency, length, content, follow-up, experience) were described.

Outcome data were organised in the following domains: (a) feasibility, (b) acceptability and usability and (c) efficacy.

Data from the RCTs were not combined due to using different comparator arms. A meta-analysis was completed instead using the pre-post study data. A meta-analysis was performed using the outcome measures reported in the studies, these were: Pain, Anxiety, Depression, Psychological wellbeing and other physical symptoms (tiredness, drowsiness, nausea, appetite and shortness of breath). The meta-analysis was performed if more than one study reported the outcome of interest, by any scale. We calculated the standardised mean differences (Hedges’s *g*) comparing the pre- and post-data scores. Statistical heterogeneity was assessed with the *I*^2^ statistic (an *I*^2^ value equal or more than 50% would have been considered as substantial heterogeneity^26^). As the patient populations were quite variable in disease type and age a DerSimonian-Laird random effects model meta-analysis was conducted using STATA version 17.0. Publication bias was assessed using funnel plots per outcome.

### Quality of the evidence

The Grading of Recommendations, Assessment, Develop-ment and Evaluations (GRADE) framework^27^ was used to assess the quality of the evidence available. The GRADE profiler (GRADEPRO) allowed us to create a summary table of the findings. Two reviewers (NW and JM) independently rated the certainty of the evidence for each domain. The evidence was downgraded by one level for serious (or by two for very serious) risk of bias, indirectness of evidence, imprecision of effect estimates or potential publication bias. Studies that were observational in design started as low quality. The quality of evidence was independently checked by a third reviewer (VV).

## Results

**Figure 1.**
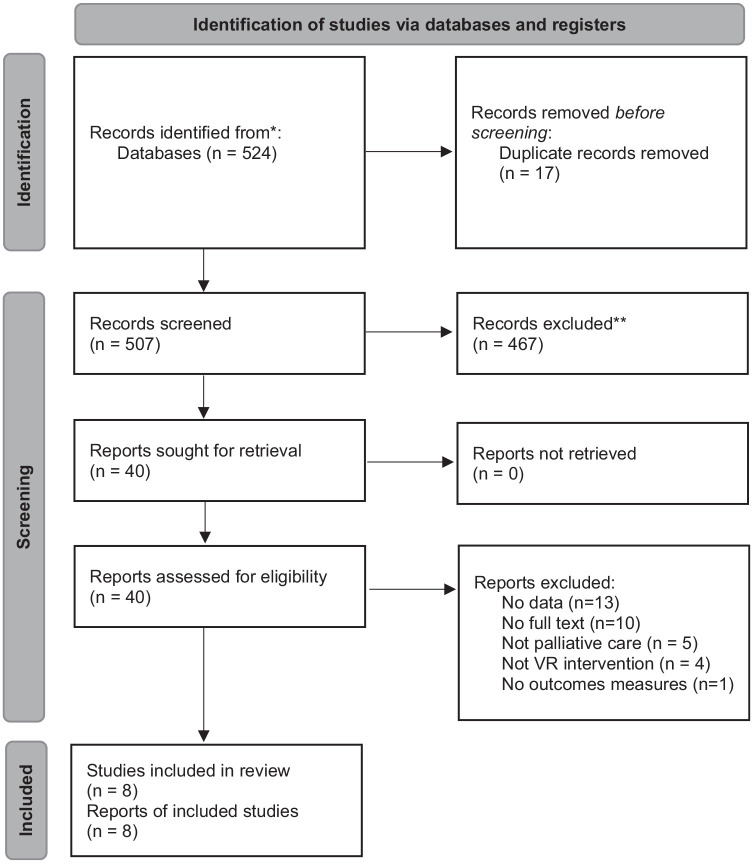
PRISMA flowchart.

A total of 524 published articles were retrieved from the database searches (See Figure 1). Following de-duplication, 507 studies were included in the title and abstract screening. Forty studies were included for full-text screening, of which 33 were excluded. After contacting the authors of abstracts and included papers, one additional paper was identified that was in press.^28^ Eight studies^28–35^ were included in the final review, of which five^28,29,33–35^ were included in a meta-analysis.

### Study characteristics

Characteristics of included studies are shown in Table 1. All studies were conducted between 2012 and 2021. Six studies^29–34^ were non-randomised studies and two studies^28,35^ were randomised controlled trials (RCTs). The RCTs compared the virtual reality to guided imagery (meditation) or to a different virtual reality experience. One study^30^ had no baseline data.

**Table 1.** Study and participant characteristics.

Study characteristics						Participant characteristics				
Authors	Country	Year	Setting	Comparator	Total Sample size (*n*)	Diagnosis		Gender		Age
								Male	Female	
							*n* (%)	*n* (%)		Mean (SD)
Baños et al.^25^	Spain	2012	Inpatient hospital	None	19	Cancer	19 (100)	10 (53)	9 (47)	60.9 (14.5)
Brungardt et al.^26^	USA	2020	Inpatient hospital	None	23	Cancer Heart failure end-stage renal	14 (61), 7 (30), 2 (9)	11 (48)	12 (52)	47.7 (17.1)
Dang et al.^27^	USA	2020	Ambulatory care unit	None	12	Cancer	12 (100)	5 (42)	7 (58)	24–65+a
Ferguson et al.^28^	USA	2020	Multiple	None	25	Dementia	25 (100)	3 (12)	22 (88)	85 (8.9)
Groninger et al.^24^	USA	2021	Inpatient hospital	Guided-imagery	88	Heart failure	88 (100)	44 (50)	44 (50)	56 (13.2)
Johnson et al.^32^	USA	2020	Hospice	None	12	Cancer heart failure, bronchiectasis, Pneumonia	8 (67), 2 (17), 1 (8), 1 (8)	4 (33)	8 (67)	72 (16)
Niki et al.^33^	Japan	2019	Palliative care wards	None	20	Cancer	20 (100)	14 (70)	6 (30)	72.3 (11.9)
Perna et al.^31^	UK	2021	Hospice inpatient	Non-personalised, VR	20	Cancer, other	15 (75), 5 (25)	6 (30)	14 (70)	66a

a Age range/Perna et al. did not report SD.

### Participant characteristics

There were 225 participants in total, with demographic data reported for 219 participants. One study^35^ only reported the demographic data of those who completed all sessions (*n* = 20/26). There were 97/219 (44%) males and 122/219 (56%) females. The mean age ranged from 47.4 to 85 years: ranging between 20 and up to 103 years of age.

In total, 3/8 (37.5%) studies included only oncology patients, 3/8 (37.5%) studies included patients with diverse types of advanced diseases, 1/8 (12.5%) studies included patients with advanced heart failure and 1/8 (12.5%) studies included only patients with dementia. See Table 1 for more detail.

### Quality appraisal

All studies had at least some concerns for risk of bias. The six non-randomised observational studies all had serious or critical risks in the domains of confounding and outcome measurements. One RCT^35^ had some concerns for bias in the domains of the randomisation process, deviations from intended interventions and outcome measurements. The remaining RCT^28^ had some concerns for bias in the domain of deviations from intended intervention (See Supplemental Material 2).

### Virtual reality intervention characteristics

Table 2 lists the characteristics of the virtual reality technology used by the studies. Two studies^28,30^ employed the same virtual reality headset, however there was no overall consistency in the technology or virtual reality platform used. Five studies (63%) adopted a 30-min single virtual reality session as the intervention,^30–34^ one study used a single 10-min virtual reality intervention session ^28^; two studies completed multiple virtual reality sessions of either four 30-min virtual reality sessions over a week^29^ or a 4-min virtual reality session once a week for 4 weeks.^35^

**Table 2.** Characteristics of virtual reality intervention.

Authors	Intervention	Comparator	Technology	Duration of treatment	Follow-up
*Randomised controlled trials*					
Groninger et al.^28^	Guided walk-in virtual environment with narration	Active control (guided imagery)	Oculus Go VR headset	One 10-min session	Same day
Perna et al.^35^	Personalised virtual reality experience based on participants preference	Non-personalised virtual reality experiences	Google Daydream headset; Google Pixel XL smartphone and headphones.	Four 4-min/week VR sessions for 4 weeks	None
*Non-randomised controlled trials*					
Baños et al.^29^	Navigation through virtual environment to induce joy and relaxation	Pre-post data	LCD screen connected to a computer; headphone, keyboard, mouse	Four 30-min sessions/1 week	4 times/week
Brungardt et al.^30^	Virtual-based music therapy with customised soundtrack	None	Oculus Go VR headset	One approx. 30-min session	Same day
Dang et al.^31^	Virtual reality-based life review using synchronised personalised avatar	Pre-post data	MoCap (Motion capture device); VocingHan hardware; Logitech wireless headset	One approx. 30-min session	1-month
Ferguson et al.^32^	Virtual reality-based 360° beach viewing	Pre-post data	Lenovo’s Mirage Solo VR headset with business edition	One 30-min session	3–5 h after invention (behavioural changes only)
Johnson et al.^33^	Virtual reality still images/animated videos viewing using one or more Virtual reality applications in Oculus Library	Pre-post data	Samsung Gear VR	One 30-min session	None
Niki et al.^34^	Virtual reality travel to the destination according to participants’ wishes	Pre-post data	VR headset HTC VIVE and VR software Google Earth VR	One 30-min session (time shortened or extended as needed)	None

### Outcomes used for virtual reality in palliative care

Table 3 summarises the outcome domains and measures reported in all included studies. All 8 (100%) studies included one or more acceptability measures of the virtual reality intervention; 5/8 (62.5%) studies reported usability measures and 4/8 (50%) reported feasibility measures; 7/8 studies (88%) reported at least one psychological and/or physical outcome measure.

**Table 3.** Specific outcomes reported and measures used.

	Authors							
	Baños et al.^29^	Brungardt et al.^30^	Dang et al.^31^	Ferguson et al.^32^	Groninger et al.^28^	Johnson et al.^33^	Niki et al.^34^	Perna et al.^35^
*Domains*								
Feasibility	✓	✓	✓					✓
Acceptability	✓	✓	✓	✓	✓	✓	✓	✓
Usability	✓	✓	✓	✓		✓		
Pain	✓		✓		✓	✓	✓	✓
Mood	✓^a^							
Anxiety	✓		✓			✓	✓	✓
Depression			✓			✓	✓	✓
Psychological wellbeing			✓			✓	✓	✓
Other physical symptoms	✓^b^		✓^d^		✓^c^	✓^d^	✓^d^	✓^d^
Othere			✓	✓	✓			

a Consisted of 7 items: joy, sadness, anxiety, relax, vigour (1 ‘not at all’ to 7 ‘completely’), general mood (scale of 1–7 where 7 was equivalent to positive mood and well-being) and subjective mood change (from −3 ‘much worse’ to +3 ‘much better’).

b Consisted of fatigue, pain and physical discomfort (0 ‘not at all’ to 10 ‘very much so’).

c Subdomains of the FACIT-Pal-14: shortness of breath, distress (0 ‘not at all’ to 4 ‘very much’).

d As measured by the ESAS-r.

e Dang et al., included measures of Health related quality of life, symptom burden and spiritual wellbeing; Ferguson et al., measured behavioural changes after the virtual reality session; Groninger et al. also measured quality of life.

Seven out of the eight included studies (88%) reported on the impact of virtual reality on physical and/or psychological domains.^28,29,31–35^ The 8 h study reported only on the acceptability and feasibility of using virtual reality^30^ using a numerical rating scale. See Table 3 for more details.

One study^32^ reported the behavioural change of the participants between 3 and 5 h after the intervention, through a qualitative interview. The remaining seven studies provided quantitative data, using the following measures: ESAS-r,^36-37^ FACIT-Pal-14,^38^ FACIT-Sp,^39^ visual analogue scales and numerical rating scales Supplemental Material 5.

### Feasibility

#### Recruitment

Available recruitment information for included studies is in Table 4. Three (37.5%) studies reported a recruitment goal,^28,31,35^ of which 2/3 (67%) reached their sample target within the recruitment period. Six (75%) studies reported a recruitment period, which ranged from 1 month up to 20 months. One study^35^ mentioned potential recruitment barriers, which was not having an assigned researcher to conduct the research.

**Table 4.** Recruitment information.

Authors	Recruitment					Retention	
	Time (months)	Target	Screened	Eligible	Consented	Rate (%)	Reasons for attrition
		*n* (%)					
*Randomised control trials*							
Groninger et al.^28^	17	128	nr	nr	94	94	nr
Perna et al.^35^	20	26	nr	26	26 (100)	77	Illness (*n* = 5), death (*n* = 1)
*Non-randomised control trials*							
Baños et al.^29^	nr	nr	nr	26	20 (77)	55	Discharge (*n* = 4), high physical discomfort (*n* = 2), presence of other worries (*n* = 1), voluntary withdrawal (*n* = 1), clinical deterioration (*n* = 1)
Brungardt et al.^30^	5	nr	33	28	23 (82)	74	Not feeling well (*n* = 3), delirium (*n* = 2), not available (*n* = 1)
Dang et al.^31^	1	12	nr	17	12 (71)	92	Did not want to talk about feelings or share stories (*n* = 1)
Ferguson et al.^32^	nr	nr	nr	nr	25	100	
Johnson et al.^33^	7	nr	nr	nr	12	100	
Niki et al.^34^	5	nr	nr	nr	20	100	

nr: not reported.

#### Retention

Among the four studies^29–31,35^ that had information on participant consent, the proportion of participants who consented to participant when approached ranged from 71% up to 100%. The proportion of participants who completed the studies ranged from 55% up to 100%. Deterioration due to ill health was one of the main reasons for leaving the trial. See Table 4 for more detail.

### Acceptability and usability

All included studies except Perna et al.^35^ included one or more general measure of participant satisfaction; with most participants reported being moderately satisfied with the virtual reality intervention. Perna et al.^35^ measured acceptability in terms of attrition data, which was surpassed (over 60% completed).

Four studies (50%)^29,30,32,33^ reported difficulties in using the virtual reality, including unfamiliarity with the software and hardware, difficulty wearing the headset at a comfortable position, difficulty making mouse movement, involuntary keyboard strokes, difficulties getting used to the button configuration of the remote controller and not able to see the image clearly. Brungardt et al.^30^ reported a mean SUS score of 80.4 (SD 13.8) suggesting that participants were happy with the usability of the virtual reality equipment.

Ferguson et al.^32^ reported that 22/25 participants had a PAINAD score of 0 at baseline and 23/25 had a PAINAD score of 0 5 min after the virtual reality experience. Four studies (50%)^29,31–33^ reported that participants experienced some discomfort using the device, including uncomfortable position, physical challenges and not getting used to wearing the virtual reality headset. One study^29^ reported that participants required the assistance of a clinician in using the virtual reality device due to symptom severity and high level of discomfort. Adverse events of the virtual reality were reported in 2/8 (25%) studies,^29,33^ including tiredness, worsening of existing dizziness and sore shoulders due to repeated adjustment of the virtual reality headset.

Six studies (75%)^28–33^ indicated that participants had positive attitudes towards the virtual reality session, perceived the intervention as beneficial were willing to repeat the intervention again or recommend to others.

### Efficacy of virtual reality in palliative care

Groninger et al.^28^ and Perna et al.^35^ were the RCTs included in this review. Groninger et al.^28^ reported that patients in both groups experienced a significant reduction in pain scores; those who completed the virtual reality session compared to the guided imagery had significantly lower pain scores (−2.9 ± 2.6 vs −1.3 ± 1.8, *p* = 0.0153). Perna et al.^35^ reported no difference in using personalised versus non-personalised virtual reality experiences.

#### Meta-analysis

Six studies reported data on the same patient outcomes ^28,29,31,33–35^; one study did not report the study data in enough detail and did not respond to an email request prior to the analysis^31^; therefore five studies were included in the meta-analysis. Four of these used the ESAS-r as the outcome measure. For this reason, we used the ESAS-r domains to structure the meta-analysis. The funnel plot (see Supplemental Material 3) indicates no evidence of publication bias.

**Figure 2.**
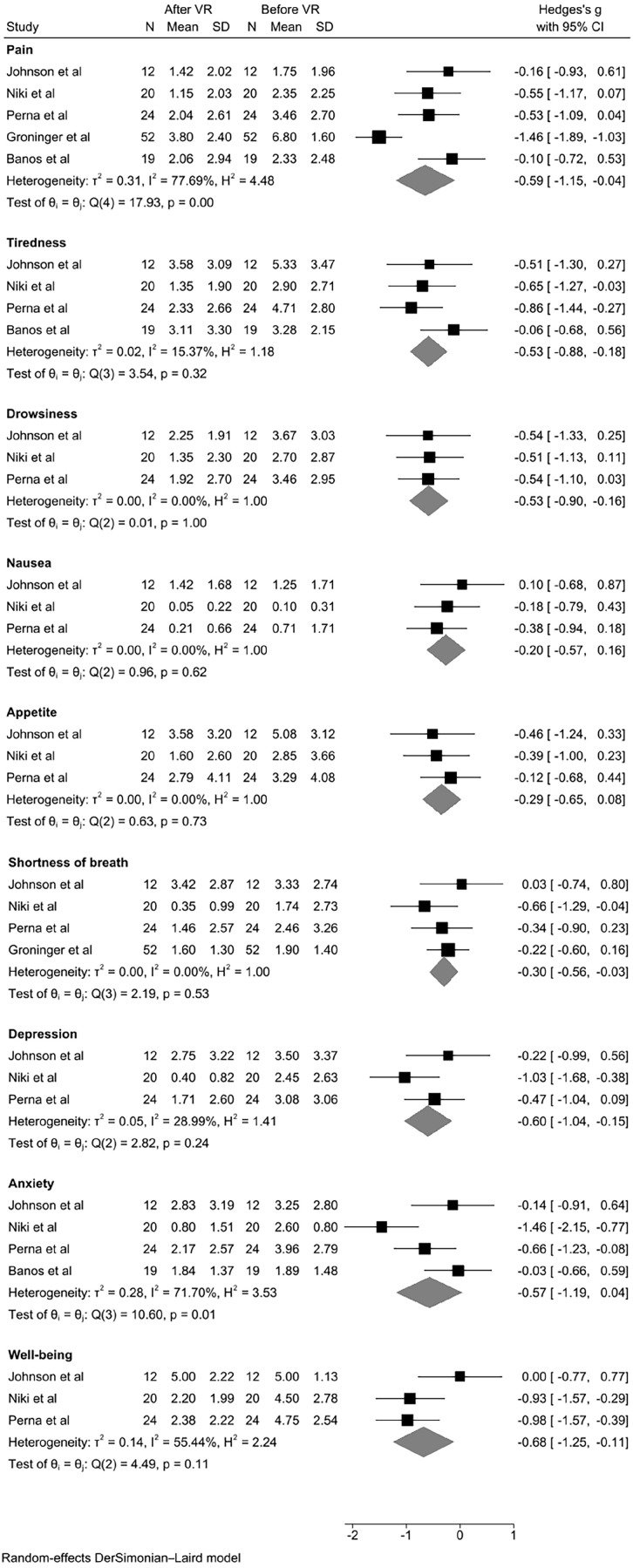
Forest plot.

Figure 2 reports the forest plot of the studies, by patient outcomes. Further meta-analyses indicated that the following domains showed significant differences between the pre- and post-data; pain (*p* = 0.0363), tiredness (*p* = 0.0030), drowsiness (*p* = 0.0051), shortness of breath (*p* = 0.0284), depression (*p* = 0.0091), psychological well-being (*p* = 0.0201).

#### Other measures

##### Spiritual wellbeing using the FACIT-Sp

Dang et al.^31^ reported that there were no significant difference pre- and post-intervention for spiritual wellbeing.

##### Quality of life

Groninger et al.^28^ reported a significant improvement in total FACIT-Pal-14 scores in both the virtual reality and guided imagery groups. Dang et al.^31^ reported no significant difference in EORTC QLQ-C30 scores.^40^

### GRADE evidence statement

Supplemental Material 5 shows the GRADE quality of evidence assessment and summary of findings. We judged the quality of the evidence for virtual reality on outcomes measured in patient outcomes as very low. Our confidence in the effect estimate is limited. We downgraded the certainty of evidence due to the risk of bias, imprecision and due to the observational design of four out of the five studies.

## Discussion

### Main findings

Findings from the studies included in this review suggest that recruitment to a virtual reality trial in palliative care was possible. It also shows that people who are living with a terminal illness enjoyed using virtual reality technology with few to no adverse reactions noted. The meta-analysis on the efficacy of virtual reality on patient outcomes suggests that there could be a therapeutic benefit to virtual reality, however the quality of the evidence was rated as low to very low due to the small sample sizes, and the study design in that that there was often no comparator arm.

### Strengths and weaknesses

This is the first rigorous systematic review to investigate the use of virtual reality in palliative care; however, there are a few areas of caution to consider when interpreting the results. Firstly, six out of the eight studies included were feasibility studies with no control group. Secondly, as virtual reality is an emerging technology, there was no agreed methodology across the studies including: the equipment used, the procedures employed (e.g. how many sessions, number of follow-ups), the type of virtual reality experience (the earliest study in 2012 used a computer to watch the experience, whereas the later studies published between 2019 and 2021 employed headsets that either had an inbuilt virtual reality experience or used a smartphone), the quality of the experience (this was often not described although one study did describe the challenge of sourcing a high quality experience from the internet^35^), and the outcomes used to measure the efficacy of the virtual reality. No study addressed the cost-effectiveness of the virtual reality compared to the efficacy. Only studies reported in English were included in this review, which could mean that some studies were omitted in other languages.

### What this study adds

This review reports the same as previous systematic reviews published looking at virtual reality in other settings; that further higher quality research is needed to offer definitive recommendations for clinical practice. A heterogeneous mix of outcome measures, study designs and virtual reality equipment limits the generalisability of the findings. No study in this review discussed capturing the efficacy of virtual reality on chronic and acute pain; only two studies completed more than one virtual reality session.^29,35^ Previous research has focussed on the impact of virtual reality on acute pain (i.e. during a procedure) however, patients under palliative care often experience chronic pain too. More research is needed to fully capture how virtual reality might best support people living with a terminal illness.

Virtual reality is an emerging technology with potential in multiple settings. It offers the opportunity for individualised care which can be readily accessed by the patient, at any time. As the technology is developing and we are becoming more familiar with using technology as part of our routine healthcare, it is vital to determine the efficacy of such methods. Additionally, if virtual reality is to become a routine part of healthcare, it is important that the appropriate policy measures are taken to ensure that the platforms and experiences are monitored for content and quality, as often the poor quality can lead to negative experiences (such as nausea or headaches).

Further research is needed to understand the efficacy of virtual reality in a palliative care setting. This review highlights the methodological and clinical challenges that need to be addressed. Methodologically, more rigorous study designs and standardised outcome measures are needed to improve the quality of the evidence. Clinically, more exploration into acute pain versus chronic pain versus disease progression within palliative care is needed to fully understand where the therapeutic benefit is of using virtual reality for people living with a terminal illness.

## Supplemental Material

sj-jpg-3-pmj-10.1177_02692163221099584 – Supplemental material for How effective is virtual reality technology in palliative care? A systematic review and meta-analysis Supplemental material, sj-jpg-3-pmj-10.1177_02692163221099584 for How effective is virtual reality technology in palliative care? A systematic review and meta-analysis by Jiping Mo, Victoria Vickerstaff, Ollie Minton, Simon Tavabie, Mark Taubert, Patrick Stone and Nicola White in Palliative Medicine

sj-jpg-4-pmj-10.1177_02692163221099584 – Supplemental material for How effective is virtual reality technology in palliative care? A systematic review and meta-analysis Supplemental material, sj-jpg-4-pmj-10.1177_02692163221099584 for How effective is virtual reality technology in palliative care? A systematic review and meta-analysis by Jiping Mo, Victoria Vickerstaff, Ollie Minton, Simon Tavabie, Mark Taubert, Patrick Stone and Nicola White in Palliative Medicine

sj-pdf-1-pmj-10.1177_02692163221099584 – Supplemental material for How effective is virtual reality technology in palliative care? A systematic review and meta-analysis Supplemental material, sj-pdf-1-pmj-10.1177_02692163221099584 for How effective is virtual reality technology in palliative care? A systematic review and meta-analysis by Jiping Mo, Victoria Vickerstaff, Ollie Minton, Simon Tavabie, Mark Taubert, Patrick Stone and Nicola White in Palliative Medicine

sj-pdf-2-pmj-10.1177_02692163221099584 – Supplemental material for How effective is virtual reality technology in palliative care? A systematic review and meta-analysis Supplemental material, sj-pdf-2-pmj-10.1177_02692163221099584 for How effective is virtual reality technology in palliative care? A systematic review and meta-analysis by Jiping Mo, Victoria Vickerstaff, Ollie Minton, Simon Tavabie, Mark Taubert, Patrick Stone and Nicola White in Palliative Medicine
