# Supplementary figures and images for: How effective is virtual reality technology in palliative care? A systematic review and meta-analysis
Source: Palliat Med. 2022 May 30;36(7):1047–58. doi: 10.1177/02692163221099584 (PMC9248003; doi:10.1177/02692163221099584)

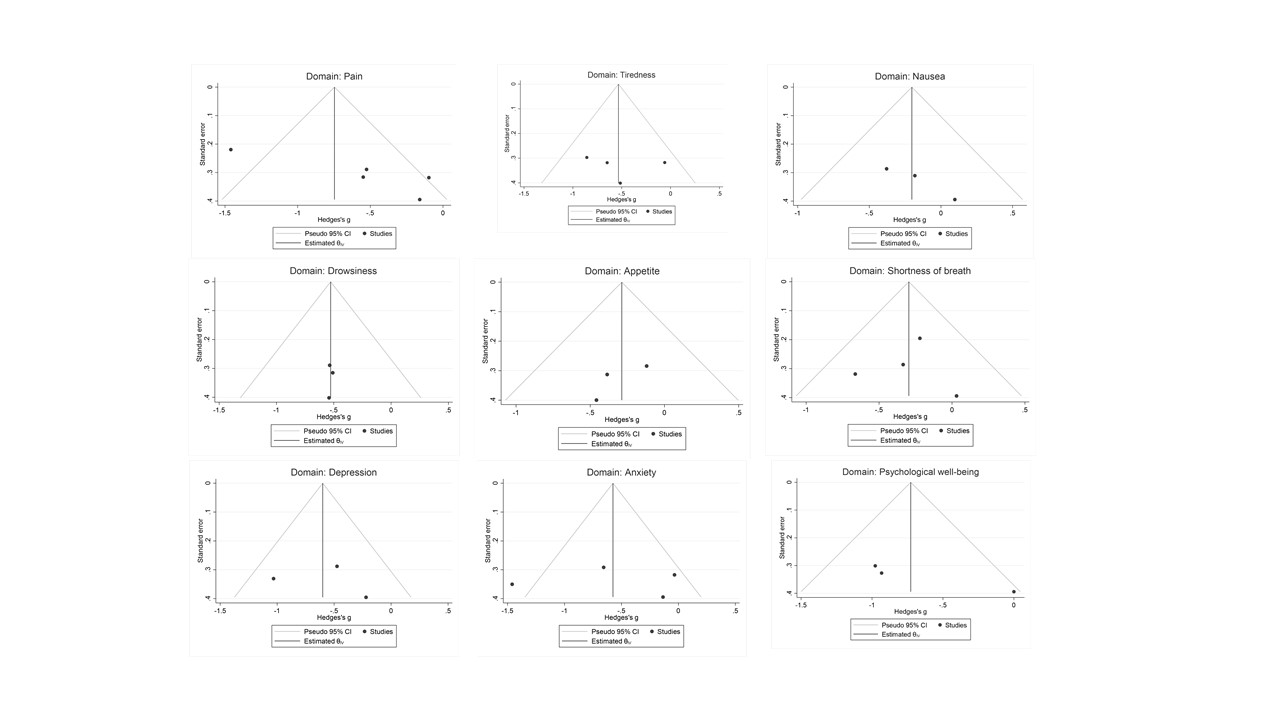

Supplement: sj-jpg-3-pmj-10.1177_02692163221099584 – Supplemental material for How effective is virtual reality technology in palliative care? A systematic review and meta-analysis [file sj-jpg-3-pmj-10.1177_02692163221099584.jpg]

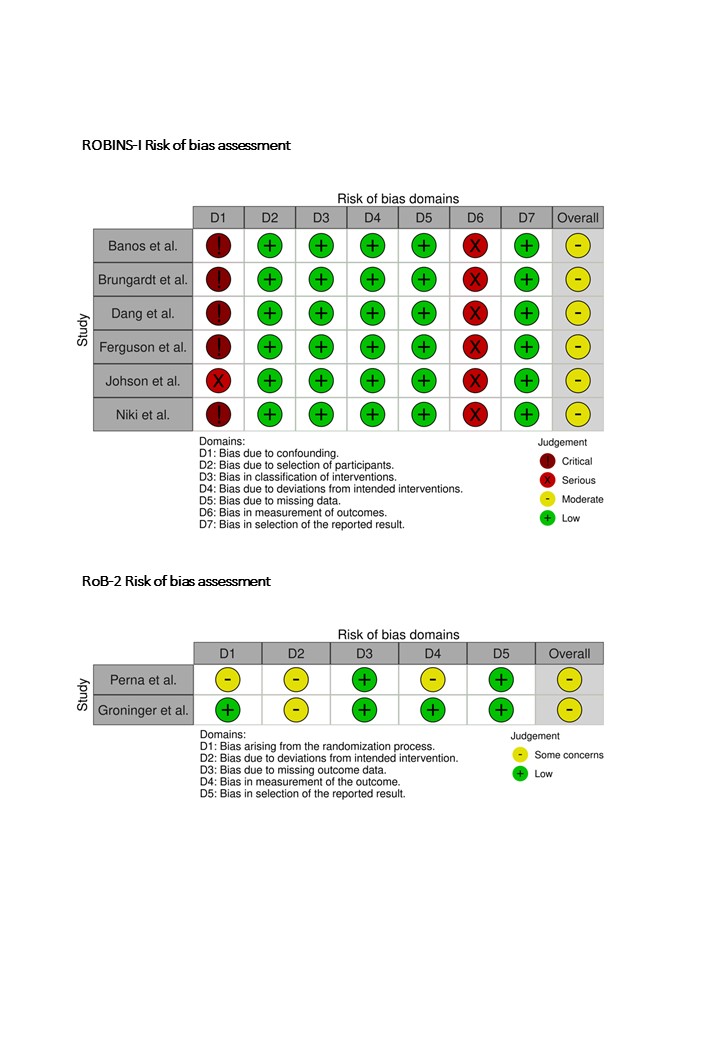

Supplement: sj-jpg-4-pmj-10.1177_02692163221099584 – Supplemental material for How effective is virtual reality technology in palliative care? A systematic review and meta-analysis [file sj-jpg-4-pmj-10.1177_02692163221099584.jpg]
