# Supplementary material for: How effective is virtual reality technology in palliative care? A systematic review and meta-analysis
Source: Palliat Med. 2022 May 30;36(7):1047–58. doi: 10.1177/02692163221099584 (PMC9248003; doi:10.1177/02692163221099584)
Supplement: sj-pdf-1-pmj-10.1177_02692163221099584 – Supplemental material for How effective is virtual reality technology in palliative care? A systematic review and meta-analysis [file sj-pdf-1-pmj-10.1177_02692163221099584.pdf]

GRADE Evidence

Certainty assessment							Summary findings				
**No. of studies**	**Design**	**Bias**	**Inconsistency**	**Indirectness**	**Imprecision**	**Other considerations**	**Sample size**		**Effect**	**Certainty**	**Importance**
							**Pre-**	**Post**	**Standardised Mean Difference (95% CI)**		
**Anxiety**											
4	Observational[a]	Serious[b]	Serious[c]	Not serious	Serious[d]		75	75	SMD 0.57 SD lower (1.19 lower to 0.04 higher)	⊕OOO VERY LOW	CRITICAL
**Depression**											
3	Observational[a]	Serious[b]	Serious[c]	Not serious	Serious[d]		56	56	SMD 0.6 SD lower (1.04 lower to 0.15 lower)	⊕OOO VERY LOW	CRITICAL
**Psychological wellbeing**											
3	Observational[a]	Serious[b]	Not serious	Not serious	Serious[d]		56	56	SMD 0.68 SD lower (1.25 lower to 0.11 lower)	⊕OOO VERY LOW	CRITICAL
**Pain**											
5	Observational and RCT[a]	Serious[b]	Serious[c]	Not serious	Serious[d]		127	127	SMD 0.59 SD lower (1.15 lower to 0.04 lower)	⊕OOO VERY LOW	CRITICAL
**Tiredness**											
4	Observational[a]	Serious[b]	Not serious	Not serious	Serious[d]		75	75	SMD 0.53 SD lower (0.88 lower to 0.18 lower)	⊕OOO VERY LOW	CRITICAL
**Drowsiness**											
3	Observational[a]	Serious[b]	Not serious	Not serious	Serious[d]		56	56	SMD 0.53 SD lower (0.9 lower to 0.16 lower)	⊕OOO VERY LOW	CRITICAL
**Nausea**											
3	Observational[a]	Serious[b]	Not serious	Not serious	Serious[d]		56	56	SMD 0.2 SD lower (0.57 lower to 0.16 lower)	⊕OOO VERY LOW	CRITICAL
**Lack of Appetite**											
3	Observational[a]	Serious[b]	Not serious	Not serious	Serious[d]		56	56	SMD 0.29 SD lower (0.65 lower to 0.08 higher)	⊕OOO VERY LOW	CRITICAL
**Shortness of Breath (SoB)**											
4	Observational and RCT[a]	Serious[b]	Not serious	Not serious	Serious[d]		106	106	SMD 0.3 SD lower (0.56 lower to 0.03 lower)	⊕OOO VERY LOW	CRITICAL

[a] Downgraded by two levels as the majority of the evidence was from studies with observational/non-randomised study design;[b] Downgraded by one level as the majority of the evidence was from studies with moderate/high risk of bias. Majority of studies only had one arm so it's not possible to control for all confounders. [c] Downgraded by one level due to inconsistency of findings reported. [d] Downgraded by one level for serious imprecision as data derived from few participants.
