# Supplementary material for: How effective is virtual reality technology in palliative care? A systematic review and meta-analysis
Source: Palliat Med. 2022 May 30;36(7):1047–58. doi: 10.1177/02692163221099584 (PMC9248003; doi:10.1177/02692163221099584)
Supplement: sj-pdf-2-pmj-10.1177_02692163221099584 – Supplemental material for How effective is virtual reality technology in palliative care? A systematic review and meta-analysis [file sj-pdf-2-pmj-10.1177_02692163221099584.pdf]

Example search strategy

Database(s): **Ovid MEDLINE(R) ALL** 1946 to March 26, 2021

Search Strategy:

#	Searches	Results
1	Palliative Care/	57814
2	terminal care/ or hospice care/	35438
3	Terminally Ill/	6679
4	palliative care.tw.	30940
5	terminal care.tw.	1541
6	hospice care.tw.	3284
7	terminally ill.tw.	5551
8	bereavement care.tw.	385
9	end of life.tw.	25078
10	advance* cancer*.tw.	14904
11	advance* tumo?r*.tw.	7401
12	palliative therap*.tw.	2288
13	1 or 2 or 3 or 4 or 5 or 6 or 7 or 8 or 9 or 10 or 11 or 12	125713
14	augmented reality/ or virtual reality/	3697
15	Ambient Intelligence/	24
16	virtual realit*.tw.	11712
17	VR.tw.	9333
18	virtual environment*.tw.	3580
19	virtual world*.tw.	789
20	virtual system*.tw.	105
21	virtual partner*.tw.	67
22	virtual interact*.tw.	162
23	virtual base*.tw.	28
24	computer simulated environment*.tw.	15
25	simulated interaction*.tw.	67
26	augment* realit*.tw.	2638
27	augment* game*.tw.	2
28	virtual game.tw.	39
29	or/14-28	22717
30	13 and 29	49
31	limit 30 to english language	47
